# *De Novo* Assembly of the Perennial Ryegrass Transcriptome Using an RNA-Seq Strategy

**DOI:** 10.1371/journal.pone.0103567

**Published:** 2014-08-15

**Authors:** Jacqueline D. Farrell, Stephen Byrne, Cristiana Paina, Torben Asp

**Affiliations:** Department of Molecular Biology and Genetics, Aarhus University, Research Centre Flakkebjerg, Slagelse, Denmark; University of North Carolina at Charlotte, United States of America

## Abstract

**Background:**

Perennial ryegrass is a highly heterozygous outbreeding grass species used for turf and forage production. Heterozygosity can affect de-Bruijn graph assembly making *de novo* transcriptome assembly of species such as perennial ryegrass challenging. Creating a reference transcriptome from a homozygous perennial ryegrass genotype can circumvent the challenge of heterozygosity. The goals of this study were to perform RNA-sequencing on multiple tissues from a highly inbred genotype to develop a reference transcriptome. This was complemented with RNA-sequencing of a highly heterozygous genotype for SNP calling.

**Result:**

*De novo* transcriptome assembly of the inbred genotype created 185,833 transcripts with an average length of 830 base pairs. Within the inbred reference transcriptome 78,560 predicted open reading frames were found of which 24,434 were predicted as complete. Functional annotation found 50,890 transcripts with a BLASTp hit from the Swiss-Prot non-redundant database, 58,941 transcripts with a Pfam protein domain and 1,151 transcripts encoding putative secreted peptides. To evaluate the reference transcriptome we targeted the high-affinity K^+^ transporter gene family and found multiple orthologs. Using the longest unique open reading frames as the reference sequence, 64,242 single nucleotide polymorphisms were found. One thousand sixty one open reading frames from the inbred genotype contained heterozygous sites, confirming the high degree of homozygosity.

**Conclusion:**

Our study has developed an annotated, comprehensive transcriptome reference for perennial ryegrass that can aid in determining genetic variation, expression analysis, genome annotation, and gene mapping.

## Introduction

High throughput sequencing technologies are now making it possible to sequence the genomes and transcriptomes of non-model plant species, many of which are of significant agricultural importance. This is particularly prevalent for transcriptomes, where improved algorithms have been developed to re-construct the transcriptome of a species *de novo*, without the assistance of a reference genome [1]–[3]. Reference transcriptomes can then be used in studies characterizing genetic variation in transcribed regions, and in global gene expression studies. Measuring transcript abundance directly through sequencing (RNA-seq) is now largely replacing hybridization based arrays [4]. The RNA-seq strategy does not rely on hybridization to a catalogue of probes on an array, and avoids problems associated with such hybridization based approaches (e.g. cross hybridization, inefficient hybridization due to polymorphism between array and sample). Problems associated with hybridization based approaches can be exacerbated by outbreeding species which contain a high degree of heterozygosity. RNA-seq is also reported to have a much greater dynamic range and sensitivity for detecting transcripts expressed at either very high or low levels [4]–[6].

Current approaches to assemble transcriptomes *de novo* from short read data are predominantly based on initially identifying contiguous sequence by creating a de-Bruijn graph of overlapping k-mers [1]–[3], [7], [8]. Factors that affect de-Bruijn graph assembly include; degree of heterozygosity, repeats in the underlying sequence, and sequencing error rate [9], [10]. Algorithms have been developed with strategies in mind to deal with these challenges. There are also challenges in re-constructing the complexity of the transcriptome, and in identifying potential splice variants. The Trinity pipeline attempts to address some of the challenges to re-construct the complexity of the transcriptome. Trinity assembles unique transcript sequence from the reads, which are then clustered together based on overlapping sequences. Each cluster is analyzed separately to reconstruct splice isoforms. During the assembly process benchmarks are in place to disregard any chimeric transcripts [1], [11].

Implementing a *de novo* transcriptome assembly using a highly heterozygous genotype complicates the assembly process and can lead to fragmented assemblies. It is envisaged that using a highly homozygous line would improve contiguity of the assembled transcripts.

Perennial ryegrass (*L. perenne* L.) is a member of the Poaceae family, which is used for turf and forage purposes in temperate agriculture [12]. It is a highly heterozygous outbreeding species with a self-incompatibility complex [13]. Currently, there are 21,898 ESTs in the NCBI database (as of February 23, 2013).

In this study the transcriptomes from multiple tissues of two perennial ryegrass genotypes were sequenced via RNA-seq. One genotype is an inbred line named P226/135/16, and has been self-pollinated for six generations. The transcriptome of the inbred genotype was sequenced in order to develop a reference suitable for expression studies, annotating genomic sequence, and anchoring genetic variation. It was anticipated that the high degree of homozygosity in this genotype would aid in *de novo* assembly. A second heterozygous genotype named F1-30 was sequenced to characterize genetic variation. F1-30 is one the parents in the VrnA mapping population, and was generated from crossing a genotype from the synthetic perennial ryegrass variety ‘Veyo’ with one genotype from the perennial ryegrass ecotype ‘Falster’ [14]. The VrnA mapping population has been used in multiple mapping studies [14]–[19]. The aim of this study was (1) to create a *de novo* reference transcriptome from an inbred genotype, (2) annotate the reference transcriptome, (3) evaluate the reference transcriptome with a well characterized gene family, high-affinity K^+^ transporters, (4) evaluate the expression profile of transcripts encoding potential signaling peptides and (5) identify SNP markers within coding regions using RNA-seq data from a highly heterozygosis genotype that is the parent of a mapping population.

## Results

### *De novo* assembly of the perennial ryegrass transcriptome

The number of reads generated for the inbred genotype was 72,132,380 pairs from six different tissues. The number of reads generated for F1-30 was 90,691,192 pairs from five different tissues. The average fragment size sequenced was less than 200 bp. Therefore, the sequence pairs have been merged into a single longer read. This yielded 36,727,657 sequences for the inbred genotype P226/135/16 with a maximum length of 232 bp and a mean length of 131 bp (std. 17.37 bp). In the case of F1-30, the heterozygous genotype, merging resulted in 31,263,328 sequences with a maximum length of 232 bp and a mean length of 119 bp (std. 14.66 bp).

This data was used to generate independent *de novo* transcriptome assemblies for each genotype using the Trinity analysis pipeline. Trinity assembled 217,162 transcripts for P226/135/16 and 198,760 transcripts for F1-30. The maximum and average lengths in both assemblies are similar. However, the P226/135/16 assembly has higher N50 and component values (Table 1). Abundance estimates of each transcript were obtained by mapping reads back to the assemblies, and transcripts with low read support were filtered out. In the case of Trinity assemblies, components loosely correspond to genes, which can have multiple transcripts that represent putative splice variants. The transcripts with low read support are likely to be assembly artifacts. Filtering removed 31,021 transcripts from P226/135/16, and 30,095 transcripts from F1-30. The filtering of the transcripts did have a significant effect on assembly statistics (Table 1). For example, in P226/135/16 we filtered out only 14.3% of the transcripts but lost 60.8 Mbp of sequence, and our N50 dropped from 1,705 bp to 1,393 bp (Table 1).

**Table 1:** *De novo* assembly summary.

	Before Abundance Estimation		After Abundance Estimation	
Metric	P226/135/16	F1-30	P226/135/16	F1-30
**Putative Transcripts**	217,162	198,760	185,833	168,665
**# of comp.**	146,810	121,152	146,559	121,144
**# of comp. with splice variants**	17,196	20,797	15,515	18,613
**# of comp. >1 kb in length**	72,709	64,780	50,029	45,362
**Maximum Length (bp)**	15,275	15,263	15,275	15,263
**Average Length (bp)**	991	953	830	824
**N50**	1,705	1,583	1,393	1,350
**Total Mbp**	215.1	189.3	154.3	138.9

**Figure 1:**
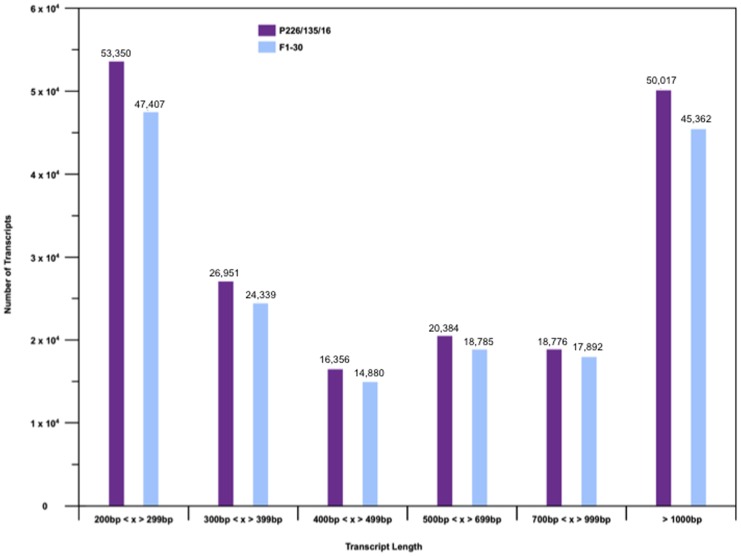
Comparison of P226/135/16 and F1-30 transcript length. A vertical bar chart of the transcript length distribution of both P226/135/16 and F1-30 transcriptomes. X axis is the ranges of transcript length in base pairs. Y axis is the number of transcripts and is a logarithmic scale.

As expected, the high degree of homozygosity in P226/135/16 enabled a more contiguous transcriptome assembly. There are more transcripts of longer length in the inbred assembly, in comparison to the assembly using reads from the heterozygous genotype (Figure 1, Table 1). A comparison of transcript lengths from our study with previous *de novo* RNA-seq studies reveals that both the inbreed P226/135/16 and the heterozygous F1-30 have a longer average length than previous studies and the N50 value is comparable with other transcriptome studies [20], [21].

The longest transcript from both P226/135/16 (15,275 bp) and F1-30 (15,263 bp) both matched (91% identity) to a predicted Brachypodium (*Brachypodium distachyon*) BIG-like auxin transport gene (13,391 bp). The BIG auxin transport gene has been shown to be involved in auxin-mediated organ growth [22] and is required for normal auxin transport [23]. This gene is one of the largest genes found in eukaryotic transcriptomes and is commonly used to evaluate the success of an assembly. The assembled transcripts from the P226/135/16 genotype have been deposited at DDBJ/EMBL/GenBank under accession GAYX00000000. The version described in this paper is the first version, GAYX01000000.

**Figure 2:**
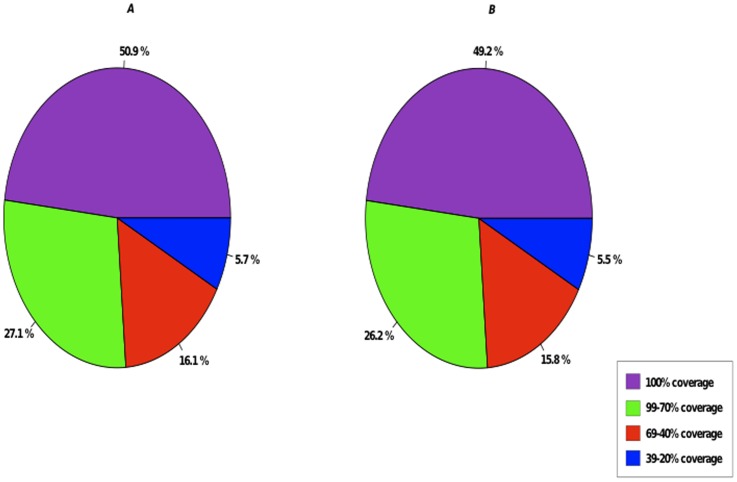
P226/135/16 assembly evaluation. (A) Percentage coverage of 15,025 Brachypodium peptide sequences to predicted perennial ryegrass peptide sequences. (B) Percentage coverage of 15,528 Rice peptide sequences to predicted perennial ryegrass peptide sequences.

To evaluate the quality of our inbred P226/135/16 reference transcriptome assembly, we took advantage of the available peptide sequences from Brachypodium and Rice (*Oryza sativa*), two species of the Poaceae family that have had their genomes sequenced. A BLASTp search was performed using the predicted peptide sequences as the query and Brachypodium or Rice peptides as the target database. We found 55,321 predicted peptide sequences with similarity to Brachypodium or Rice peptide sequences. Using the 32,255 sequences from Brachypodium, we discovered that the P226/135/16 had a 20% hit coverage or higher with 15,025 Brachypodium peptide sequences. In the comparison with the 66,338 Rice peptide sequences, P226/135/16 had a 20% hit coverage or higher with 15,528 sequences. Over seven thousand peptide sequences from both Brachypodium and Rice had a hit coverage of 100% (Figure 2).

### Functional annotation of the inbred perennial ryegrass transcriptome

**Figure 3:**
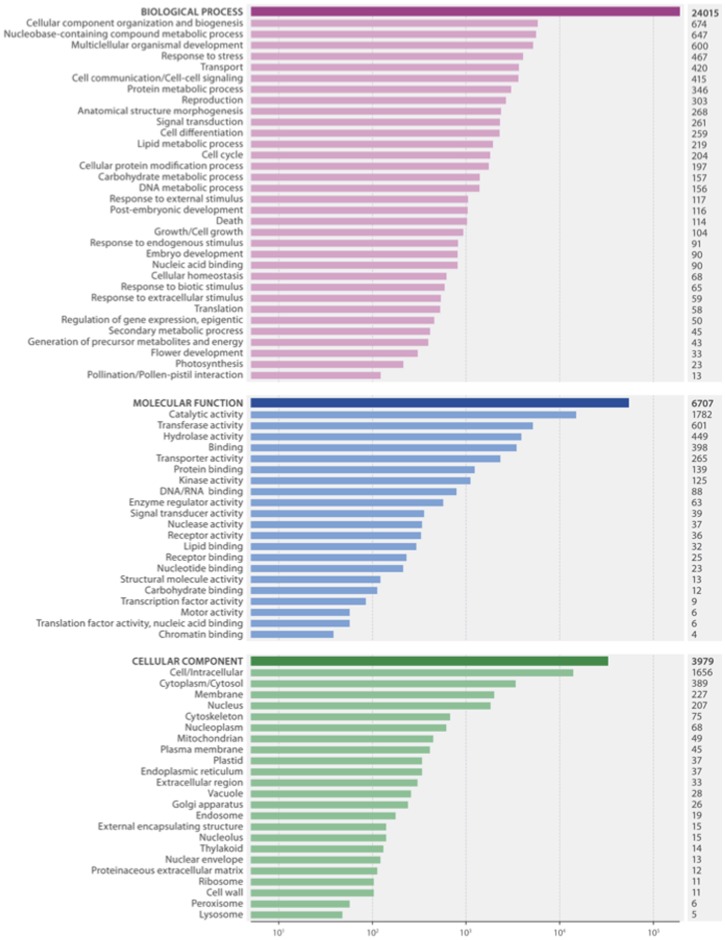
Plant GO-Slim terms associated with inbred perennial ryegrass reference transcriptome. Horizontal bar chart of the distribution of plant GO slim terms associated with the inbred genotype P226/135/16 transcripts represented in biological process, cellular component and molecular function categories. X axis is a logarithmic scale.

We predicted 78,560 peptide sequences of which 24,434 were predicted to be complete. Approximately 51,000 of the transcripts with a predicted peptide sequence also had a significant BLASTx with the Swiss-Prot NR database (Table 2; Table S1). The transcripts without a BLASTx match could be from an untranslated region, non-coding RNA, correspond to genes without any annotation information and/or lack a protein domain [20], [24]. Over 75% of the predicted peptide sequences were found to contain a Pfam protein domain; 15% contained transmembrane helices and 4.8% were found to contain a predicted signal peptide (Table 2; Table S1). A majority of the predicted signal peptides (61.8%) did not contain transmembrane helix, indicating that these could be potential signal peptides for secreted proteins [25]. To determine if our reference transcriptome contained a wide range of biological, cellular and molecular functions typical of a plant transcriptome, we mapped gene ontology (GO) terms to a plant GO slim file [26]. The distribution of transcripts within these GO categories (Figure 3) resembles similar transcriptome assemblies [24], [27]–[29].

**Table 2:** Overview of Functional annotation output of inbred P226/135/16 reference transcriptome.

Database	Number of transcripts
**Swiss Prot (NR)**	50,890
**Pfam**	58,941
**Signal Peptide**	3,811
**Transmembrane helices**	11,800
**Eggnog**	41,348
**Gene Ontology**	48,790

### Single nucleotide polymorphism discovery

We used the nucleotide sequence of the 52,315 longest predicted open reading frames (ORFs) as the reference sequence for single nucleotide polymorphism (SNP) discovery. Reads from the inbred and heterozygous genotypes were mapped back to the reference for SNP calling. Using our criteria, 64,242 SNPs were found in 19,568 predicted ORFs. The majority of the SNPs discovered were polymorphic between genotypes (Table 3; Table S1).

**Table 3:** Summary of SNP discovery data.

	SNPs	SNPs/1 kb	# of ORFs
**SNPs between genotypes**	36,084	1.4	13,855
**P226/135/16**			
Total SNPs	2,667	0.1	1,061
Synonymous SNPs	1,704	0.06	802
Non-synonymous SNPs	963	0.04	580
**F1-30**			
Total SNPs	26,789	1	11,410
Synonymous SNPs	19,436	0.7	9,447
Non-synonymous SNPs	7,353	0.3	5,046

Within the inbred P226/135/16 genotype we discovered 2,667 SNPs, approximately one SNP per ten kb. These SNPs were found in 1,061 predicted ORFs of which the majority are annotated. Of the 2,667 SNPs found, over 64% were synonymous (Table 3; Table S1). We discovered 26,789 SNPs in the heterozygous F1-30 genotype, approximately one SNP per one kb. Similar to P226/135/16, the majority (73%) of the SNPs found in F1-30 were synonymous (Table 3; Table S1).

The majority (69%) of the SNPs were synonymous substitutions. As shown in previous research [30], [31], 98% of the synonymous SNPs were found in the third base of the codon. The remaining 8,316 SNPs found were non-synonymous (NS) substitutions with 87% NS SNPs found in the first or second base of the codon.

### Putative ryegrass High-affinity K^+^ Transporter genes

**Figure 4:**
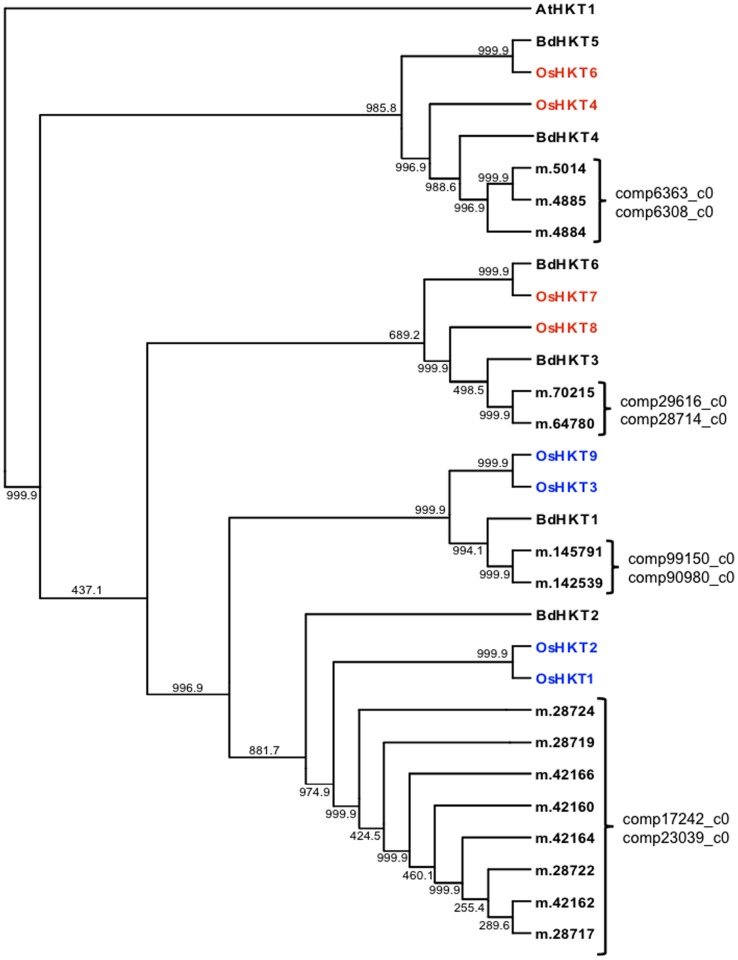
Parsimony tree of HKT gene family. Peptide sequences from Rice (OsHKT), Arabidopsis (AtHKT), Brachypodium (BdHKT) and predicted HKT peptide sequences from perianal ryegrass. Component names associate with predicted peptide sequences are after the bracket (}). OsHKT labels in red are classified as subfamily 1, OsHKT labels in blue are classified as subfamily 2. Bootstrap values are shown at the nodes.

To further evaluate our reference assembly we looked at a gene family of importance in grasses in order to determine if we could identify all orthologs in our assembly. Using the high-affinity K^+^ transporter (HKT) gene family peptide sequences from the model species Rice (OsHKT) and Brachypodium (BdHKT), we were able to identify one or more predicted peptide sequence for each of the HKT query sequences (Figure 4, Table S2). A total of fifteen predicted peptide sequences show high similarity to OsHKT and BdHKT peptide sequences and all but two of the predicted perennial ryegrass peptide sequences are predicted to be complete. We used the GUIDANCE server [32] and PHYLIP [33] to cluster the genes based on peptide similarity (Figure 4, Table S2). The grouping of the OsHKT genes is similar to that shown in previous studies [34], [35]. The grouping of the BdHKT genes in relation to the OsHKT genes agrees with a previous study [36].

There is one HKT gene in Arabidopsis (*Arabidopsis thaliana*) and nine in Rice. In Rice one HKT gene (OsHKT5) is a pseudogene and was not used in this evaluation [37]. In Brachypodium there are six putative HKT genes [36]. We found putative orthologs for all eight OsHKT genes in the ryegrass reference transcriptome (Figure 4, Table S2). Fifteen peptide sequences from eight different components were found to have high similarity to the Rice and Brachypodium HKT peptide sequences. None of the fifteen P226/135/16 ORFs with similarity with OsHKT and BdHKT genes contained any SNPs.

### Expression profile of predicted secreted peptides

**Figure 5:**
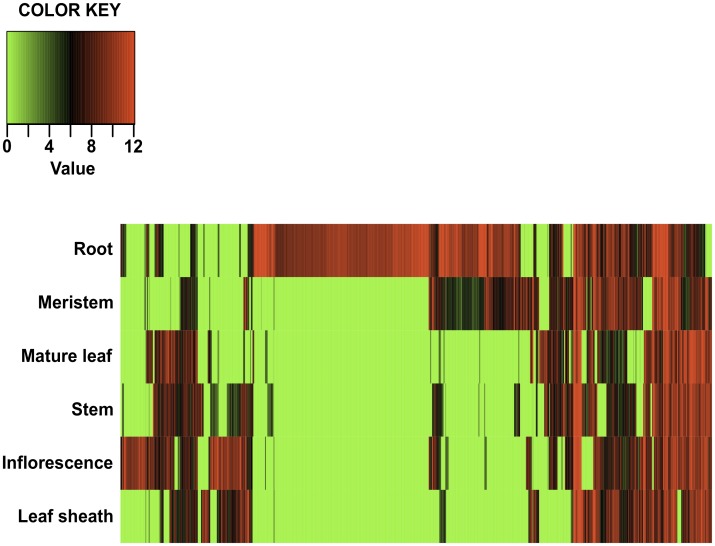
Heat map of predicted secreted signaling peptide expression in six tissues. Each row represents a predicted secreted signaling peptide, while each column represents the six tissues used in this study. The color key represents the median centered log2 TMM-normalized FPKM values. Red indicates a high level of expression, black indicates no change in expression, and green indicates low level of expression.

**Figure 6:**
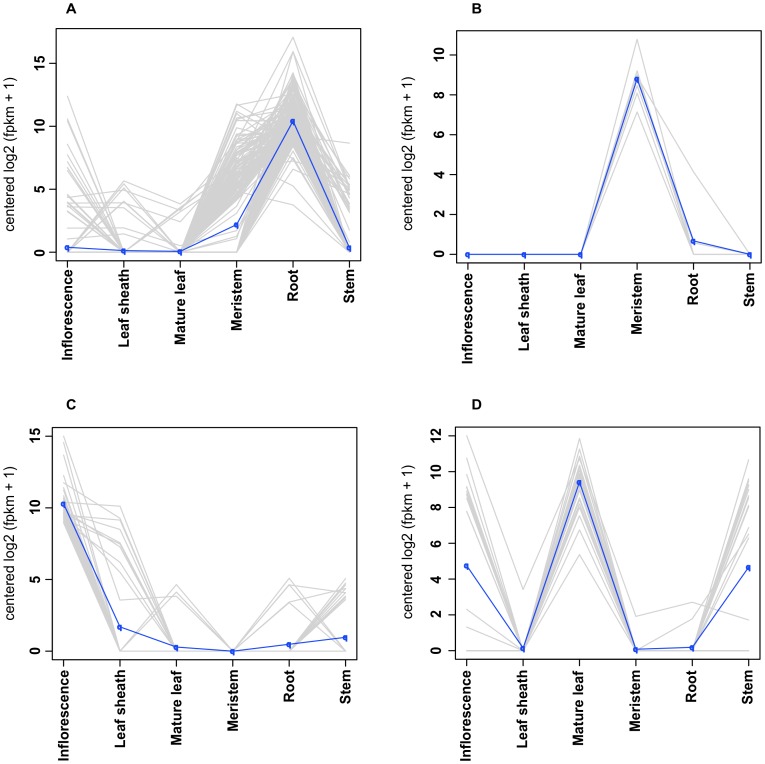
Expression profiles of differentially expressed predicted signaling peptides. The X-axis is the six tissues and the Y-axis is the median centered log2 expression. (A) 314 transcripts show high expression in root tissue. (B) 7 transcripts show high expression in meristem tissue. (C) 44 transcripts show high expression in both inflorescence tissue, (D) 24 transcripts show high expression in inflorescence, mature leaf, and stem tissue.

By identifying transcripts containing a signal peptide, amino acid length shorter than 250 and lacking a transmembrane domain we were able to predict 1,151 transcripts encoding predicted secreted peptides (Figure S1). We were able to determine if any of the predicted secreted peptides had a tissue specific expression profile (Figure 5). Out of the 1,151 transcripts encoding predicted secreted peptides 712 (62%) were found to be differentially expressed (False Discovery Rate (FDR) ≤0.001) in different tissues. To determine which cellular, molecular or biological functions the differentially expressed transcripts encoding secreted peptides contained, we mapped GO terms to a plant GO slim file [26]. The majority of the functions associated with the differentially expressed transcripts encoding predicted secreted peptides were categorized as biological process (53%) (Figure S2). Of the 712 differentially expressed transcripts encoding predicted secreted peptides (Figure 5) a large portion (314 transcripts) are found in the root (Figure 5A). Past research has shown that secreted peptides play an important role in root stem cells [38], and root development [39]. We also observed 44 transcripts encoding putative secreted peptides with differential expression in the inflorescence (Figure 5, Figure 6B). Seven transcripts encoding predicted secreted peptides were found to be highly expressed in the meristem (Figure 6C). Twenty-four transcripts encoding predicted secreted peptides showed high to moderate expression in the inflorescence, mature leaf and stem (Figure 6D).

## Discussion

Next generation sequencing (NGS) is now enabling genomics to be applied to non-model species. Both the transcriptomes and genomes of these organisms can now be studied in a manner that was previously only possible for a limited number of model species. Recently, there has been a plethora of studies reporting *de novo* transcriptome assemblies from short read data in many non-model organisms [21], [40]–[47]. This has been made possible by advancements in NGS technology and improvements in computational algorithms for de *novo* assembly [1]–[3]. In this study we have generated a reference transcriptome for the important forage and turf grass, perennial ryegrass and the assembled transcripts have been deposited in public databases.

### Assembly and annotation of the inbred perennial ryegrass reference transcriptome

We report a *de novo* transcriptome assembly of a highly inbred perennial ryegrass genotype using Illumina RNA-seq reads. A paper has been recently published introducing a procedure, Orthology Guided Assembly (OGA), to develop a reference transcriptome from highly heterozygous crop species [31]. In developing the procedure the authors used short read data from 14 highly heterozygous perennial ryegrass genotypes and by implementing the OGA method were able to generate a reference transcriptome to uncover the genetic diversity between genotypes [31]. They identified 19,345 non-redundant coding sequences and the sequence data is available on request from the authors. This approach captured gene families with high homology to Brachypodium, and may not have captured the full complexity of the perennial ryegrass transcriptome. However, this strategy proved to be a very useful approach to capture genetic variation within the species.

The highly heterozygous perennial ryegrass genotypes served as an excellent resource to identify genetic variation within the assembled transcripts [31]. We believe our reference assembly complements the OGA study in a number of ways. Our reference assembly is based on sequencing the transcriptome of a highly inbred genotype. This greatly simplifies assembly and allows re-construction of transcripts that would otherwise be fragmented due to high heterozygosity (Figure 1, Table 1). Furthermore, our assembly is based on RNA sourced from six plant tissues, which may allow recovery of a broader spectrum of transcripts. We were able to assemble 186,141 transcripts with an N50 of 1,393 kb and a mean length of 0.83 kb. These assembly statistics are in line with previous assemblies [20], [21], [27], [48]. Our inbred transcriptome assembly has been deposited into the NCBI TSA.

### Secreted peptide expression profile

Being a sessile organism, plants respond to their changing environment by a unique signaling system. Signaling peptides, transcription factors and auxin are used to communicate between cells within the plant [49]. Peptides with an N-terminal signal peptide, no transmembrane domain, and less than 250 amino acids in length are putatively secreted signal peptides [25]. Secreted peptides are extracellular compounds, which act in an autonomous manner between adjacent cells [49]. Using computational methods over 1000 putative secreted peptides where found in the Arabidopsis genome. To date only ten of these secreted peptides have been matched to a receptor or functionally characterized [39], [50]. Secreted peptides have been shown to play an important role in plant development [51], pollen-pistil interactions [52], cell-cell communication [50] and root stem cell maintenance [38], [39].

We were able to identify 1,151 putative secreted peptides in our transcriptome database. After mapping the gene ontology terms from the putative secreted peptides to the plant GO slim file we found a wide range of categories as see in Figure 3 (Figure S1). As we had sequenced RNA from multiple tissues we were able to get an insight into the expression profiles of these putative secreted proteins. This enabled us to identify a cluster of secreted peptides highly expressed in root (Figure 5, Figure 6A, Figure S3). The presence of highly expressed secreted peptides in the root is in general agreement with previous work [38], [39], [53], [54]. It was found that the secreted peptides expressed in the root play an important role in regulating root apical meristem cells, triggering pathogen resistance and immune responses [38], [39]. Using complex positive promotion and negative feedback loops signal peptides play a key role in signal cascades within plant cells.

The presence of highly expressed secreted peptides in other tissues (inflorescence, meristem mature leaf and stem) could be involved in cell-cell communication [39], [50], plant development [51], and immune or pathogen resistance [39], [49]. Within a plant species secreted peptides families are highly diverse and can evolve rapidly. Secreted peptides form a complex intercellular signal cascade which plays a key role in plant growth, defense, and maintenance [39].

### Putative ryegrass High-affinity K^+^ Transporter (HKT) genes

Soil salinity is reducing current agricultural production and hampering the expansion of agriculture. Soil salinization is increasing worldwide due to accumulation of soluble salts from irrigation and land clearing [55]. The High-affinity K^+^ Transporter (HKT) gene family is the primary mechanism within plants for managing salt tolerance. HKT transporters minimize salt uptake and disperse the salt so it does not build up to toxic levels [56]. Improving the salt tolerance of grasses would be a significant benefit to agriculture, and with this in mind, we aimed to identify the complement of HKT genes in perennial ryegrass.

There is one HKT gene in Arabidopsis, identified to have a role in Na^+^ uptake [57]. Other cereals including Barley, Rice and wheat vary in copy number of individual HKT gene family members [58]. There are nine HKT genes found in Rice but the number of functional HKT genes varies between rice cultivars [34], [59], [60]. Within Barley, eight orthologs to Rice HKT genes were identified, and the number of putative Wheat HKT genes differs between genomes [58]. The Brachypodium genome also has five to six HKT-like genes with sequence similarity to OsHKT genes [36], [61].

When looking for othologs of the HKT family in perennial ryegrass we used Rice HKT genes as a reference because it is a model species for research in the HKT gene family. We also used Brachypodium since it is the most closely related grass species to perennial ryegrass [62]. We discovered fifteen perennial ryegrass predicted peptide sequences with high similarity to HKT genes (Figure 4, Table S2). Thirteen of the predicted peptide sequences were predicted as complete. The HKT gene family is divided into two subfamily groups based on peptide sequence and functionality [35], and we were able to clearly differentiate between the two HKT subfamily members (Figure 4, Table S2). Grouping is based on a glycine/serine substitution within the first pore loop of the HKT protein. Furthermore, functional analysis indicates that the amino acid substitution has a role in K^+^ and Na^+^ selectivity [59], [63], [64]. Rice OsHKT4-8 genes are designated to subfamily 1 which have a serine residue and considered Na^+^ specific transporter genes [34], [58]. However, there are exceptions to this general rule of thought. OsHKT1 is grouped with subfamily 2 but has a serine residue [34]. Suggesting that other elements besides amino acid residue plays a role in ion selectivity. However, with little structural knowledge and no resolved 3D structures it is difficult to predict motifs responsible for ion selectivity [61]. These putative orthologous peptide sequences in ryegrass originate from eight different components, indicating the presence of eight HKT orthologs in the reference transcriptome. Annotation of these fifteen predicted peptide sequences supports putative HKT gene function. All fifteen have a BLASTx hit with an OsHKT gene and the same cation transport protein Pfam domain.

The HKT genes from Rice and Brachypodium cluster into four relatively distinct groups. This is in general agreement with previous phylogenetic analysis of the HKT family in these two species [34]–[36], [61]. Encouragingly, we find perennial ryegrass transcripts from our assembly in all four clusters. This demonstrates the value of the transcriptome assembly for gene family analysis. The High-affinity K^+^ Transporter (HKT) gene family has an important role in salt tolerance in crop species. Interest in HKT transporters is increasing in plant biology, agronomy and plant breeding disciples [36], [56], [58], [65]. With the advancements in transcriptome and genome sequencing, there is now greater potential to study beyond the model species.

### Single nucleotide polymorphism discovery

Single nucleotide polymorphisms (SNPs) are the most common type of genetic variation in plant genomes, and can be readily discovered and characterized using sequence data [66]. Gene-associated SNPs have been used to generate genetic maps [18] and determine genetic diversity [31], [67]. RNA-seq targets SNP discovery on coding sequences and enables discovery of gene-associated SNPs [68]. For species without a reference genome, using RNA-seq to identify SNPs can be a very efficient approach in identifying genetic markers [69]. However, without a very high quality reference genome it can be difficult to call SNPs due paralogous genes. When using genomic re-sequencing strategies we can account for this to some extent by looking at the read depth. We can flag regions with very high coverage that may be indicative of duplicated regions. This is difficult with SNP calling using RNA-seq data as the coverage level will be dependent on the expression of that gene. Furthermore, we may bias our SNP discovery process to genes with high expression levels. Despite this, transcriptome re-sequencing does provide a means to reduce the complexity of the genome for SNP discovery and focus discovery on the transcribed portion of the genome.

In the reference transcriptome from the inbred genotype P226/135/16, 78,560 transcripts have a predicted protein (Table 1;). By generating 52,315 reference sequences from the longest predicted ORFs, we were able to generate a database of SNPs. Less than half of the predicted ORFs contained a polymorphism, generating a SNP frequency per predicted ORF of 3.3. The lack of allelic variations within the inbred P226/135/16 genotype shows how the six generations of self-pollination have greatly reduced the heterozygosity within the P226/135/16 genotype.

The future usefulness of any SNPs in this database will be dependent on whether the SNP segregates in the material under study. The real power will be using the inbred transcriptome as a reference for SNP calling to target expressed genes (as demonstrated here). There are multiple high throughput approaches developed to characterize variation without a reference sequence, however SNP calling is simplified with a good reference sequence.

## Conclusions

Our study has developed an annotated comprehensive transcriptome reference for perennial ryegrass. We have demonstrated the usefulness of this reference by using it to identify perennial ryegrass othologs of the HKT gene family in Rice and Brachypodium. We successfully identified fifteen transcripts from eight genes and there was a gene represented for each group within the Rice HKT gene family. The usefulness of the transcriptome reference was further demonstrated by the identification of putative secreted signal peptides, many of which have high expression in root tissue. This assembly has greatly improved the amount of sequence data available in public databases that can aid in determining genetic variation, expression analysis, genome annotation, and gene mapping.

## Materials and Methods

### RNA preparation and sequencing

Six different tissue types were collected from the perennial ryegrass genotype P226/135/16; inflorescence, leaf sheath, mature leaf, meristem, root and stem. Five tissue types were collected from the perennial ryegrass genotype F1-30; inflorescence, leaf sheath, mature leaf, meristem, and stem. A second meristem tissue sample was collected from F1-30 to generate six libraries for each genotype.

Total RNA was extracted from each sample using the RNeasy Plant Mini Kit following the manufactures instructions (Qiagen, Valencia, CA) and the RNA integrity was measured with an RNA 6000 Nano Labchip on the Agilent 2100 Bioanalyzer (Agilent Technologies, Santa Clara, CA). Using the mRNA-Seq Sample Prep Kit (Illumina, protocol version 1004898 Rev. D, September 2009), the mRNA-Seq library was generated using 10 µg total RNA from each of the twelve samples; it was purified using poly-T oligonucleotide-attached magnetic beads and fragmented using divalent cations under elevated temperature. Using reverse transcriptase and random hexamer primers, first strand cDNA copies of the mRNA fragments were generated. The second strand cDNA was then synthesized using DNA Polymerase I and RNaseH. Using the Multiplexing Sample Preparation Oliogonucleotide Kit, the Illumina adaptors were added by ligation and a fragment size of approximately 200 bp was isolated via gel purification. The libraries generated were enriched by 18 PCR cycles. Using the Invitrogen Qubit Fluorometer (Life Technologies, Carlsbad, CA), the concentrations of the libraries were determined, and purity and size of the libraries were measured using the DNA 1000 kit on the Agilent 2100 Bioanalyzer (Agilent Technologies, Santa Clara, CA). An equimolar amount of each library was pooled and diluted with EB buffer (Qiagen, Valencia, CA) to 10 nM for preparation for paired-end Illumina GAIIx sequencing.

### Initial data processing

Initial analysis showed that the fragment size of the paired-end libraries was shorter than twice the read length, meaning that on average there was an overlap between paired-end sequence one and paired-end sequence two. This was the case for both P226/135/16 and F1-30 libraries, which had been generated side by side. In genome assembly, these short fragment libraries are a requirement for the use of the AllPaths-LG [70] as they enable a larger k-mer size to be used in construction of the de-Bruijn graph. We used the error-correction model and pair merging/filling algorithm within AllPaths-LG (ErrorCorrectReads.pl). This performed error-correction and merging of pairs by recruiting additional reads to support the merging of an overlapping or near overlapping pair. The raw P226/135/16 sequencing reads were submitted to the NCBI Short Read Archive (SRA) (SRX464954).

### *De novo* transcriptome assembly

The merged and error corrected reads from each genotype were assembled independently using the Trinity pipeline (release 2013-02-25) with the following settings; Inchworm: -K –L 25; Chrysalis: -min_glue 2 -glue_factor 0.05 -min_iso_ratio 0.05 -kk 48 –strand -report_welds -max_mem_reads 1000000. The *de novo* assembled transcripts were then used as a reference to map back the individual reads and estimating abundance using RSEM [71]. We then filtered out any transcripts with less than 1% of the per-component expression level (IsoPct) using a script bundled with Trinity. These transcripts with low support are likely to be transcript assembly artifacts. ORFs were extracted using the perl script bundled with Trinity (transcripts_to_best_scoring_ORFs.pl). To estimate the number of full length transcripts that had been assembled in our data sets we used the P226/135/16 assembly as the query in a BLASTp search (evalue of −10) with the available peptide sequences from two different model plant species, Brachypodium (Brachypodium.org, Release V1, Bradi_1.0.pep.fa; Last modified Dec 2010) and Rice (MSU Rice Genome Annotation Project Release 7; Last modified Oct 2011), as the target. Using pipeline perl scripts within Trinity, we determined the percent hit coverage for the top matching target sequences [11].

### Functional annotation of the perennial ryegrass reference transcriptome

Functional annotation and analysis of the P226/135/16 *de novo* transcriptome was conducted using the Trinotate pipeline [11]. BLASTp search was performed using P226/135/16 predicted ORFs as the query and the SwissProt non-redundant database (accessed 29^th^ July 2013) as the target [72]. The HMMER and Pfam databases [73], [74] were used to predict protein domains, SignalP 4.1 [75] server was used to predict the presence of signal peptides, and the TMHMM server v2.0 [76] was used to predict transmembrane helices within the predicted ORFs from the P226/135/16 transcriptome. All the transcriptome annotation was loaded into a SQLite database. CateGOrizer [26] (version 3.218) was used to map GO terms to a parent plant GO Slim file in order to get a broad overview of the functional classification of the transcripts.

### Single nucleotide polymorphism discovery

The longest ORF from each unique Trinity component was chosen as a reference for SNP discovery. Reads from the P226/135/16 inbred genotype and the heterozygous genotype F1-30 were mapped onto the reference using BWA [77]. PicardTools was used to generate a sorted bam file. Samtools was used to generate a mpileup file with the following setting: -q 20 [78]. VarScan [79] was used to document variant calls within and between the inbreed and heterozygous genotype using the mpileup2snp feature. SNP calls were made using the following thresholds: minimum read coverage at a site of 5, and a minimum number of reads supporting the variant of 2. To call a homozygote within a genotype, a base needed to be supported by at least 80% of the reads. Perl scripts were written to filter the VarScan output and categorize variants according to polymorphism type.

### High-affinity K^+^ Transporter (HKT) gene family

High-affinity K^+^ Transporter (HKT) protein sequences of Rice and Brachypodium were used in a BLASTp search (evalue −10) against the P226/135/16 predicted proteins database. Perennial ryegrass sequences with similarity to HKT genes were chosen for phylogenetic analysis. The protein sequence alignments and confidence scores were obtained using the GUIDANCE server [32], choosing MAFFT (Multiple Alignment using Fast Fourier Transform) as sequence alignment method. Columns with confidence scores below 0.93 were removed from the alignment. The phylogenetic trees were constructed using PHYLIP software, version 3.69 [33]. As a first step, Seqboot module of the software was run with 1000 bootstrap repeats, followed by parsimony calculations using the Protpars module of the software, searching for the best tree within 1000 data sets. The Arabidopsis HKT sequence was set as outgroup. The final bootstrapped tree was created using Consense. Drawgram was used to visualize the final tree.

### Expression analysis of putative secreted peptides

We identified transcripts with predicted signal peptides, that were shorter than 250 amino acids in length and lacking a predicted transmembrane helix [25]. Using these transcripts as the reference we estimated abundance of each transcript in the different tissues by mapping reads from each tissue independently. We used RSEM [71] and edgeR [80] together with Trinity utilities to get an insight into differential expression (determined with an FDR of 0.001) of these putative secreted signal peptides in different plant tissues. The GO terms associated with each putative secreted protein were mapped to a plant GO slim file using CateGOrizer to determine broader functional classification [26] (version 3.218).

## Supporting Information

Figure S1 **Plant GO slim terms associated with predicted secreted signal peptides.** Horizontal bar chart of the distribution of plant GO slim terms associated with the 1,151 predicted secreted signal peptides represented in biological process, cellular component and molecular function categories. X axis is a logarithmic scale.
(TIFF)

Figure S2 **Plant GO slim terms associated with the differentially expressed secreted signal peptides.** Horizontal bar chart of the distribution of plant GO slim terms associated with the 712 differentially expressed transcripts represented in cellular component, molecular function and biological process categories. X axis is a logarithmic scale.
(TIFF)

Figure S3 **Plant GO slim terms associated with the differentially expressed secreted signal peptides in root tissue.** Horizontal bar chart of the distribution of plant GO slim terms associated with the 314 differentially expressed transcripts represented in cellular component, molecular function and biological process categories. X axis is a logarithmic scale.
(TIFF)

Table S1 **A tab delimited file containing the functional annotation information for the transcripts with predicted ORFs.** The information includes top BLAST hit, predicted protein domain, predicted signal peptide, gene ontology, protein sequence and SNP discovery information.
(7Z) Click here for additional data file.

Table S2 **Perennial ryegrass inbred transcriptome components and predicted peptide sequence with sequence similarity to OsHKT and BdHKT.**
(DOCX)
