# Supplementary figures and images for: De Novo Assembly of the Perennial Ryegrass Transcriptome Using an RNA-Seq Strategy
Source: PLoS One. 2014 Aug 15;9(8):e103567. doi: 10.1371/journal.pone.0103567 (PMC4134189; doi:10.1371/journal.pone.0103567)

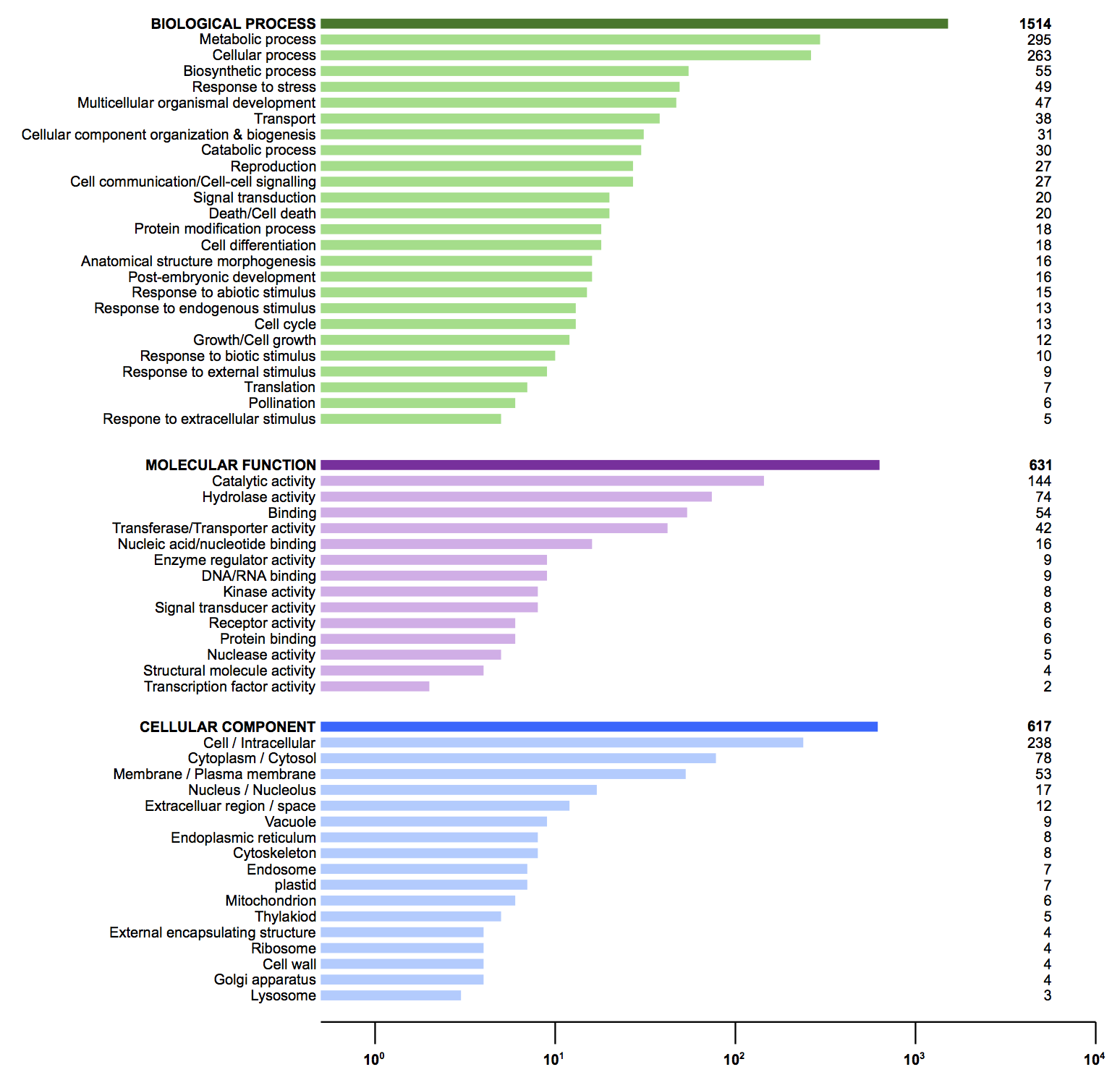

Supplement: Figure S1 — Plant GO slim terms associated with predicted secreted signal peptides. Horizontal bar chart of the distribution of plant GO slim terms associated with the 1,151 predicted secreted signal peptides represented in biological process, cellular component and molecular function categories. X axis is a logarithmic scale. (TIFF) [file pone.0103567.s001.tiff]

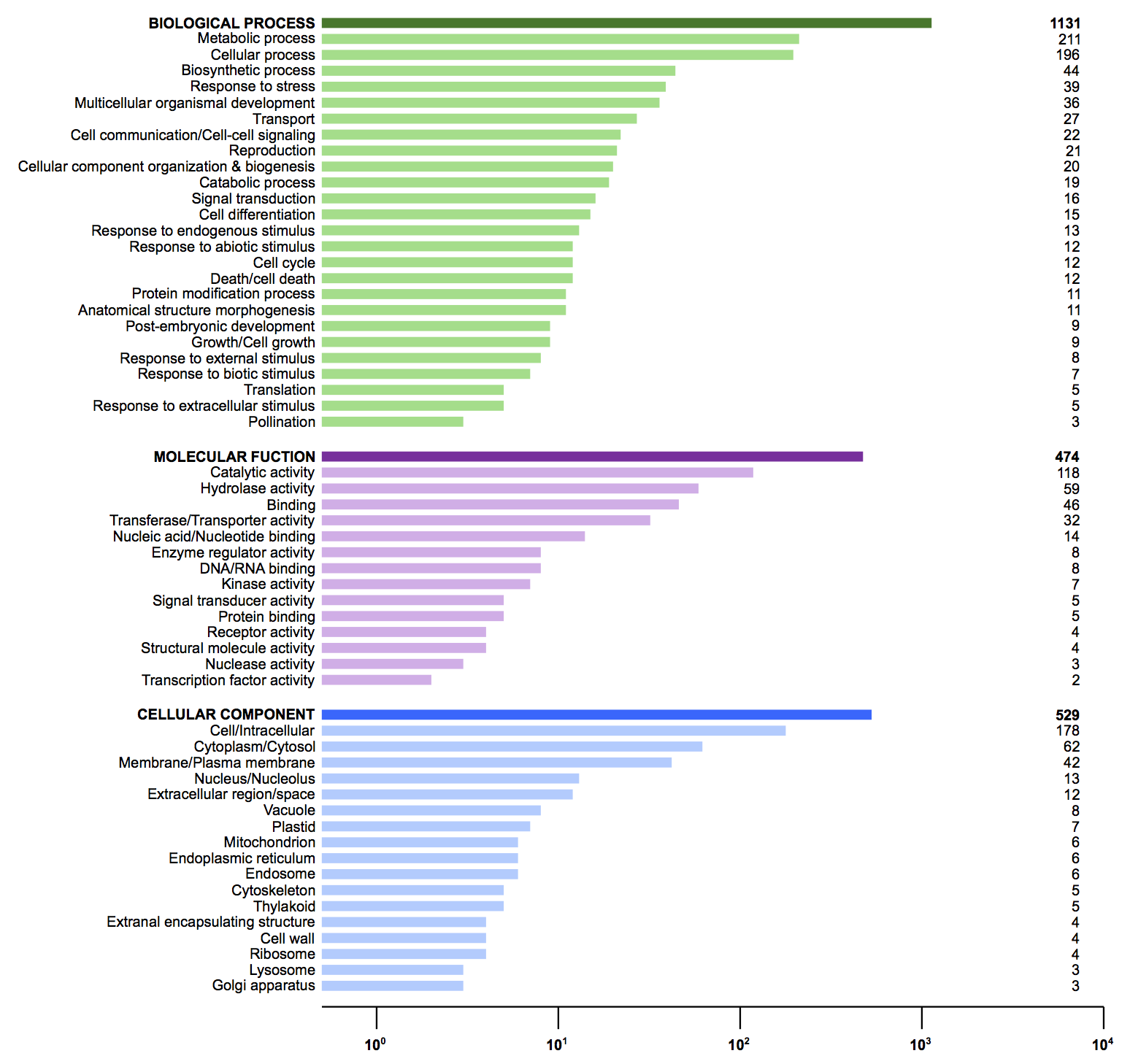

Supplement: Figure S2 — Plant GO slim terms associated with the differentially expressed secreted signal peptides. Horizontal bar chart of the distribution of plant GO slim terms associated with the 712 differentially expressed transcripts represented in cellular component, molecular function and biological process categories. X axis is a logarithmic scale. (TIFF) [file pone.0103567.s002.tiff]

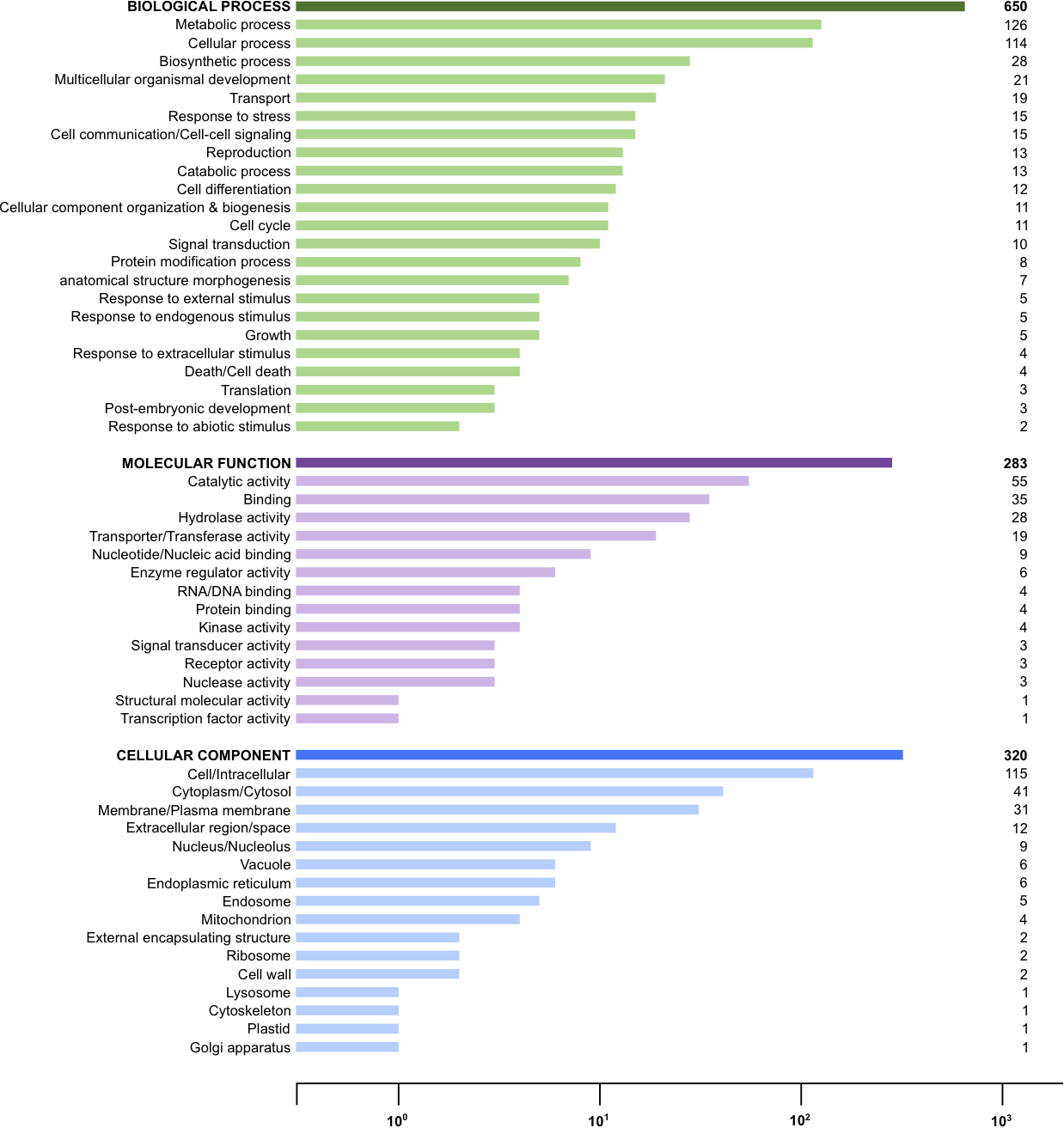

Supplement: Figure S3 — Plant GO slim terms associated with the differentially expressed secreted signal peptides in root tissue. Horizontal bar chart of the distribution of plant GO slim terms associated with the 314 differentially expressed transcripts represented in cellular component, molecular function and biological process categories. X axis is a logarithmic scale. (TIFF) [file pone.0103567.s003.tiff]
