# Supplementary material for: De Novo Assembly of the Perennial Ryegrass Transcriptome Using an RNA-Seq Strategy
Source: PLoS One. 2014 Aug 15;9(8):e103567. doi: 10.1371/journal.pone.0103567 (PMC4134189; doi:10.1371/journal.pone.0103567)
Supplement: Table S2 — Perennial ryegrass inbred transcriptome components and predicted peptide sequence with sequence similarity to OsHKT and BdHKT. (DOCX) [file pone.0103567.s005.docx]

| **Predicted peptide sequence with sequence similarity** | **Corresponding components** |
| --- | --- |
| m.5014 | comp6363_c0 |
| m.4885 | comp6308_c0 |
| m.4884 | comp6308_c0 |
| m.70215 | comp29616_c0 |
| m.64780 | comp28714_c0 |
| m.145791 | comp99150_c0 |
| m.142539 | comp90980_c0 |
| m.28724 | comp17242_c0 |
| m.28719 | comp17242_c0 |
| m.42166 | comp23039_c0 |
| m.42160 | comp23039_c0 |
| m.42164 | comp23039_c0 |
| m.28722 | comp17242_c0 |
| m.42162 | comp23039_c0 |
| m.28717 | comp17242_c0 |
